# Humanizing care in the ICU: implementation of the Get to Know Me Board in patient care

**DOI:** 10.3389/fmed.2026.1796720

**Published:** 2026-03-31

**Authors:** Sumera R. Ahmad, Amelia Barwise, Lori Rhudy, Mahmut Ozkan, Ognjen Gajic, Lioudmila V. Karnatovskaia

**Affiliations:** 1 Division of Pulmonary Critical Care, Allergy, and Sleep Medicine, Mayo Clinic, Rochester, MN, United States; 2 Biomedical Ethics Research Program, Mayo Clinic, Rochester, MN, United States; 3 Department of Graduate Nursing, Winona State University, Rochester, MN, United States; 4 Department of Internal Medicine, Jersey Shore Medical Center, Neptune City, NJ, United States

**Keywords:** clinician perspective, communication, get to know me board, humanizing, ICU - intensive care unit

## Abstract

**Introduction:**

Patient dehumanization when caring for critically ill patients in the intensive care unit (ICU) is commonplace. The Get to Know Me board (GTKMB) has been identified as a valuable tool for promoting humanized care and communication in the ICU; however, its implementation is highly variable. This study aimed to identify the barriers and facilitators influencing consistent implementation of the GTKMB in the ICU.

**Materials and methods:**

We conducted multidisciplinary focus groups via teleconference with ICU clinicians at a large quaternary care academic medical center. A thematic content analysis was performed to identify key themes and concepts related to GTKMB implementation.

**Results:**

Thirty-eight clinicians participated across six focus groups, including 10 nurses, 7 physicians, 6 advanced practice providers, 5 rehabilitation therapists (allied health clinicians), 1 respiratory therapist and 1 social worker. Major themes related to barriers and facilitators of GTKMB implementation included, (1) identifying appropriate patients and optimal timing for completing the GTKMB, (2) logistical challenges such as access to the board, visibility and unclear responsibility for completion, (3) the importance of clinician role modeling supporting the board completion, and (4) opportunities to improve the design and functionality of the board itself.

**Conclusion:**

Although clinicians recognize the GTKMB as a meaningful tool to support humanized care in the ICU, inconsistent implementation limits its potential impact. This study highlights several key barriers and facilitators to its use. Future efforts should focus on targeted quality improvement initiatives to enhance consistent completion and integration of the GTKMB into ICU practice.

## Introduction

The experience of critical illness often results in an inability to communicate, loss of autonomy, pain, and profound feelings of fear and loneliness. These factors can contribute to the loss of human attributes, a phenomenon commonly referred to as *dehumanization* (1–4). Dehumanization poses a significant threat within the ICU care processes, regardless of patient survival, as it undermines trust, disrupts communication, damages relationships between patients, families, and care teams, and may hinder recovery (5, 6). Despite these consequences such experiences are common in the fast paced and stressful ICU environment. Families have long expressed concerns regarding insufficient respect in ICU settings, where patients may be voiceless, subjected to invasive procedures, and left feeling isolated or disoriented (7–10). Both patients and families consistently underscore the importance of being recognized and treated as individuals rather than solely as medical diagnoses (11–14).

Fortunately, several approaches to promote humanized care in the ICU exist including the Get to Know Me board (GTKMB). The GTKMB is a tool designed to introduce the patient in the context of their everyday life. It originated in 2006 in the United States’ palliative care practice as a means to preserve patient’s personhood at end of life (15, 16). The GTKMB was identified as essential to the care experience of one survivor with a disability and supported another survivor in developing a recovery checklist (17, 18). During the COVID-19 pandemic, critical illness survivors reported that the GTKMB was valuable in fostering humanized care and strengthening relationships with clinicians (19). The GTKMB has been available at our institution for use in practice for at least over a decade. ICU clinicians have found the GTKMB helpful not only in humanizing care but fostering communication between patients, families and clinicians and guiding care process (20). However, the GTKMB is not a consistent part of care processes in the ICU as clinicians manage multiple competing priorities. Additionally, unlike the ICU interventions for which positive outcomes can be seen as patients recover, the lack of standardized outcomes for potentially humanizing tools like the GTKMB limit systematic uptake.

Therefore, this paper aims to describe perspectives of multidisciplinary ICU clinicians regarding barriers to implementing the GTKMB and propose strategies to support its more consistent integration into critical care practice.

## Methods

### Study setting and design

**Figure 1:**
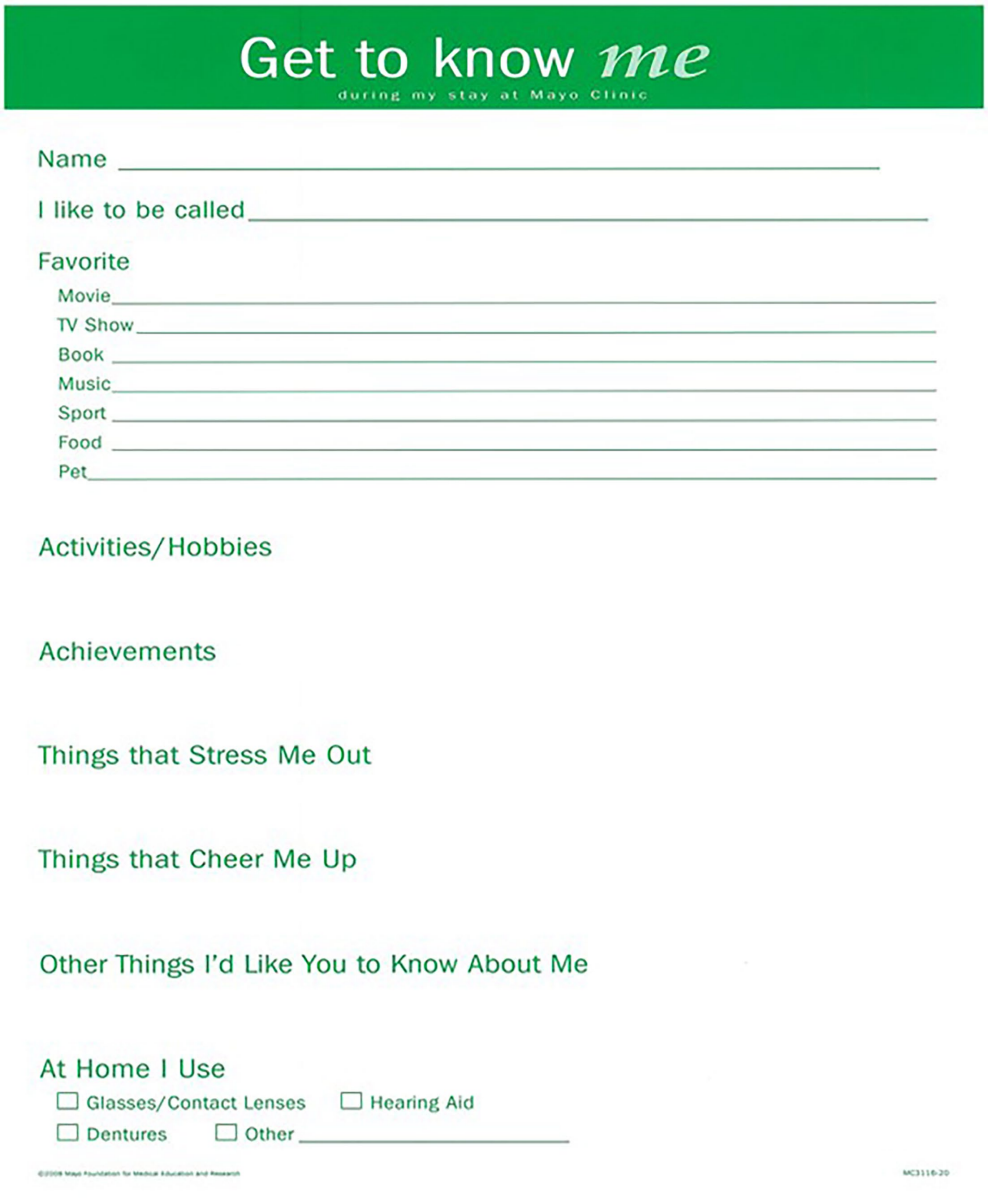
Get to know me board.

The GTKMB currently exists in our institution in a poster format to complete and introduce a patient as a person (Figure 1). Briefly, this is a qualitative study using focus group discussions, conducted at the Mayo Clinic, Rochester, a quaternary care academic medical center, where the study protocol was reviewed and deemed exempt by the Institutional Review Board (19-012799). This is a separate section of a larger study where study methods and findings related to the perceived benefits of the GTKMB have been described in detail previously (20). Because the study results were clearly reflective of two unique components—benefits of use and implementation—we chose to present the results in two separate reports.

The study is reported in accordance with the Consolidated Criteria for Reporting Qualitative Research (COREQ) guidelines (21).

### Participants and recruitment

As previously reported (20), ICU clinicians were invited to participate through email invitations sent to unit and departmental distribution lists, as well as through personal outreach, word-of-mouth invitations, and participant referrals (snowball sampling). Patients and families were not part of this analysis since their perspectives were described in a previous study using surveys and structured interviews (19).

### Data collection

The interview guide was developed by our multidisciplinary study team (LR, AB, MO, SA), which included a biomedical ethics and health care disparities researcher, a nurse scientist, and ICU physician researchers, and was informed by prior research. The guide incorporated questions derived from literature review as well as the team’s content expertise and clinical experience. In addition to questions addressing humanizing care in the ICU—the primary purpose of the study—participants were asked to describe perceived barriers to GTKMB use, suggest strategies for its optimal use, and propose any potential modifications. Questions were intentionally left open-ended to facilitate participants sharing perspectives and experiences.

Focus groups were conducted between February and April 2023. Virtual focus groups were held via videoconferencing using Microsoft Teams and facilitated by LR using a semi-structured moderator guide (Appendix S1). MO and/or AB/SA also attended the sessions and took field notes. Oral consent was obtained from all participants at the time of the focus group. Each discussion was scheduled for up to 60 min and audio-recorded, with transcripts generated by the Microsoft Teams videoconferencing software. Data collection was discontinued once data thematic saturation was reached, with no new themes emerging during the discussions (22).

### Data analysis

Data was analyzed using thematic analysis. Members of the study team independently reviewed and open-coded an initial set of two randomly selected transcripts. Insights from this process, together with field notes, were used to develop a preliminary codebook. The codebook was subsequently applied to all transcripts, which were coded independently and in duplicate by AB and LR who both have several years’ experience conducting qualitative research. The study team (including SA and MO) also met regularly to review coding decisions, resolve discrepancies, and achieve consensus. Emerging themes were identified, labeled, and examined for their relationships and organization in accordance with a general inductive approach (23). Finally, the study team met to develop overarching themes and select representative exemplar quotes.

## Results

A total of 38 ICU care providers participated in the six focus groups. Participant characteristics are described in Table 1. Participants included 10 registered nurses, 7 physicians, 6 advanced practice providers (nurse practitioners and physician assistants), 1 social worker, 5 physical and occupational therapists, and 1 respiratory therapist.

**Table 1:** Participant background in each focus group.

Focus group	Number of participants (38)	Participant background in ICU	Years of experience (range)
FG 1	4	Surgical ICU nurses	1.5–2.5
FG 2	6	Med- surg ICU nurse	<1–15
FG 3	7	2 PT, 3 OT, 1 RT, 1 SW	1.5–13
FG 4	8	Physicians: medical, surgical, med- surg ICUs	1–31
FG 5	6	Affiliate practitioners: medical/surgical/med- surg ICUs	1.5–14
FG 6	7	Physicians: medical/surgical/ med- surg ICUs	2–20

FG, Focus group; PT, physical therapist; OT, occupational therapist; RT, respiratory therapist; SW, social worker.

Four main themes emerged related to factors that influence adoption of the GTKMB. These included: (1) Right time, right patient; (2) Barriers to using the GTKMB; (3) Facilitators to using the GTKMB, and (4) Opportunities to optimize the GTKMB. See Table 2.

**Table 2:** Factors influencing implementation of the GTKMB.

Themes	Representative quotes
Right time, right patient	
Time and place-not during emergent situation or admission	There’s a time and a place where they get the get to know me board. Some people want to fill it out as soon as they get to the ICU, and I find that to be kind of burdensome, not only because we need to land the patient and do everything, but the patient and the family are also stressed. And then we ask questions about what their pets are. But once the dust kind of settles and things are kind of into a groove, I think filling it out is helpful in that regard. (6,4)
	When this is the first time they have [family] seen them [patient] intubated and sedated, and they have never seen their loved one like this, that is not the right time to do the get to know me board…maybe for some families it is, but I imagine for me if I was shocked that is not the right time. I want you to be asking about relevant information relating to their care. (5,5)
Patient characteristics- patient unable to speak, prolonged ICU admission	It seems that there is a really specific patient that you can do this with. It’s going to be the patient that is intubated, probably going to be intubated on the unit for a longer time. They have family that’s going to be involved in their care that’s willing to fill out the board. (1,4)
	It does take a specific patient, like if it’s somebody that we know is going to be a long haul… (1,3)
	Especially with our lower functioning patients or patients who do not have family that can be present at the bedside, I find the get to know me board to be extremely helpful to just at least allow us to see the person and who they were prior to hospital admission in some light so that we can start with making connections, especially when they are unable to communicate. (3,1 PT)
Barriers to implementation	
Poor visibility and accessibility	That’s maybe why we have better success with filling them out is that we stick them out right where people can see them. (2,6)
	I do not see the GTKMB like when I float or anything unless it’s like a long-term patient, I rarely see it, so I do not know if like providers are exposed to it enough to ask for it. (1,1)
	I know that we stocked the paper copies. But I would say like the embedded ICU’s I see them often use a little bit more than maybe some of the other ICU. So, I think it kind of varies maybe use and accessibility for different units. (3,2 OT)
	And I think there’s a blank one sitting in every room. Like, prior to the coming in. So, like we as nurses do not have to go get it. Like when someone sets up a room like it’s in there. (2,3)
	We have them on one of our whiteboards that are magnets. So, they stick them up there. But there’s not necessarily a pen right there or a marker and there’s not any invitation to fill them out… and this is one thing that they can do that can help all of us and give them something to do that’s useful and worthwhile…
Ambiguous responsibilities about who fills the GTKMB	Think it’s always a question of who should be filling it out. I think what was highlighted about having families know that they can also participate with them and give them something to do is I think a really important thing to identify here.
	I do not necessarily like filling out the sheet because then to me from their perspective it would be to me perceived as Oh, this is just this is just something that they have to do. And then it completely degrades the intention of the whole thing. (2,6)
	Instead of asking the family or the patient to fill it, I like to fill it myself while I’m talking to the patient or to the family, which is to me helpful in terms that you know, I’m not asking them to fill in another questionnaire, which they have done a lot of time, but it is a conversation. (6,1)
	There’s not really a consistent way in which it gets filled out…I think once it’s filled out, we all have a tendency to read it when we are going in to talk to the patients or examining the patients or at least, I do. (4,1)
Facilitators of the GTKMB	
Role modeling	It’s probably even more valuable for, let us say, the interns or residents who are a little shell shocked by the ICU environment and who are very focused on objective things, lab data and making sure that they present all this … flood of information. It helps to sort of just bring it back to the patient a little bit and like the overall. (6,5)
	… from the trainee point of view, if they should be involved in filling it out. I do not think it should be a requirement, but I think it should be encouraged that they, the residents and fellows, are there as they fill it out. (4,2)
Opportunities to optimize the GTKMB	
Format changes to support ease of completion/be more inclusive culturally and in other ways	Different cultural needs might be interesting to add to that too. Like for example, we have had multiple patients who like Native American heritage that have certain like I do not know what the appropriate word is, but like things that they want to keep in there like amulet type, things that they keep next to their bed or one patient in particular like needed like any hair that fell out of like her head or that was brushed out of her head to be like collected like certain. And I do not know if that’s necessarily generally applicable, but like certain things culturally that might be applicable as well. (3,1 PT)
	It would be helpful for me to quickly be able to look at who their family members are like. Do they have a spouse or significant other or like children but also for patients that do not have a lot of family that could be like kind of a reminder of that being alone. (3,2 OT)
	I am often talking with our ICU patients about what brings them comfort. And so did they have any comfort items that can be brought by family that we could recreate in the ICU … I’ve heard some people have had, like a rosary or a piece of jewelry or things like that, that they wanted to close to their bed or hold on to at certain times. (3,4 SW)
	That’s where I think I usually see people listing names of children and grandchildren or under achievements … Interesting that we have pet on here and not their children and grandchildren. It could be something like who supports me …. “Who is important to me”? (6,6)
	“Something I’m proud” of is a lot better than achievement’s, cause a lot of people do not fill out the achievement part. (3,5 RT)
Process change recommendations	If we could ask these questions like upon admission like prior to surgery or something … like carry it over to the room, that would make it so much easier. … If we have someone that’s intubated and sedated for, … four or five days like. (1,3)
	Maybe a kind of a routine setup would be helpful…You know, like we do not fill it out on every single patient, that’s for sure. (5,5)
	But some of these bigger procedures (surgeries) that are multiple hours, some of these basic questions could be answered for us (prior to admission). They’re not really extensive, you know, to answer it would be helpful. Just to maybe have some of that stuff, I do not know how that would be implemented, but that would be helpful. (1,4)
	Getting patients to do it ahead of time as part of their, you know clinic screenings when they come in for something elective could be helpful but then you know when you get patients who aren’t as engaged or interested in filling these surveys out, those are also sometimes the patients who we would benefit more from having it … Getting it done ahead of time as much as possible. (4,8)
	We could have liked the paper version hanging up, but then there would also be a version on the iPad on the unit or something that they could make choices. Filling boxes that type of thing. (3,4)

### Identifying the right patient at the right time

It emerged as a key factor in successful GTKMB implementation. Subthemes included considerations of timing and setting, patient characteristics, and the perceived benefit of the GTKMB. Participants emphasized the importance of sensitivity when introducing the GTKMB, particularly with respect to timing. Factors influencing timing included the need to prioritize acute care during critical illness, such as resuscitation and emergency management, as well as gauge family readiness in moments involving the delivery of terminal diagnosis. Although participants generally agreed that all patients could benefit from care informed by the GTMKB, they identified specific patient population for whom the tool may be particularly valuable, including patients who are unable to communicate for themselves, those with prolonged stays, and those without family present at bedside. Participants also emphasize that the GTKMB should complement, rather than replace, authentic clinician-patient or clinician-family conversations.

*“I think the more complex, longer staying patients benefit from it greatly … especially patients who are very critically ill, cannot speak for themselves, maybe ventilated or just not able to converse because of their cognition.” (3,3 PT)*.

### Barriers to GTKMB implementation

These included limited visibility and accessibility. Participants who worked across multiple ICUs reported substantial variability in GTKMB placement, which can affect how frequently the tool was used in certain units. They noted that GTKMB use was more consistent in units where the board was stored in highly visible and easily accessible location. Additionally, workflows for moving the GTKMB from storage to the patient room varied across units; some incorporated the GTKMB into initial room setup, various others relied on individual clinicians to locate and initiate its use.

Another barrier was ambiguity regarding roles and responsibilities for completing the GTKMB. Balancing completion of the GTKMB with clinical tasks can be stressful, particularly for newer nursing staff. Participants expressed concerns about the time required for healthcare providers to complete the board and noted the absence of writing materials. Despite the uncertainty around responsibility, participants consistently described a shared sense of ownership and collective responsibility for using the GTKMB.

*“With the new nurses, it’s being able to focus on getting these tasks done, paying attention to hemodynamics, stuff like that, rather than being able to also focus on filling out a board and paying attention to the more humanistic aspects of their care. They just do not have that time management just yet.”* (1,4).

*“I think the get to know me board would be super helpful if we just had it in a more visible area.”* (1,2).

### Facilitators for GTKMB implementation

These included role- modeling its use for novices and trainees. Participants noted that the culture and expectations surrounding humanizing care and GTKMB use were strongly shaped by preceptors, team members, and personal clinical experiences. Given the highly technical nature of the ICU environment, with its emphasis on technology and quantitative data such as hemodynamics and laboratory values, incorporating humanistic element of care was viewed as an important counterbalance. Participants emphasized the importance of role modeling and explicitly setting expectations for GTKMB use, noting that effective implementation requires deliberate and intentional strategies, such as referencing the board during team rounds. Engaging family members in completing the GTKMB was described as preferred practice and perceived to further support implementation.

*“Rounds is when we really role model to our learners and if we did try to humanize our patients a bit more, that’s a good way to role model that to our younger learners.”* (4,7).

*“Give it to the families to fill out, if the patient cannot write or talk or communicate with us, because obviously the families know and we do not know them.”* (2,3).

### Several opportunities to optimize the GTKMB

The opportunities were identified. Most participants agreed that the current content of the GTKMB was adequate. A recurring recommendation was to include comfort items that reflect culturally significant actions or personal belongings. Goal setting was seen as an important function of the GTKMB; however, incorporating this information could be controversial, particularly for patients unable to speak for themselves.

Additional suggestions focused on process improvements, such as enabling patients to provide GTKMB information prior to admission for planned surgical procedures with an anticipated ICU stay. Making the GTKMB more portable could allow its use across the continuum of care and to support transitions in care. Another recommendation included integrating the GTKMB into the electronic health record (EHR) and having paper copies that are easily transferrable across levels of care.

*“That’s where I think I usually see people listing names of children and grandchildren or under achievements … Interesting that we have pet on here and not their children and grandchildren. It could be something like who supports me … ‘Who is important to me’?”* (6,6).

## Discussion

This qualitative study used focus groups to explore ICU interprofessional team members’ perspectives on factors that facilitate or hinder the implementation of the GTKMB as a strategy to promote humanized care for ICU patients. Multidisciplinary ICU clinicians reported that the GTKMB was valuable in in fostering communication among patients, families, and clinicians, guiding care, and building connection with families (20). Despite its perceived benefits, several barriers to implementation were identified. Participants highlighted specific strategies to enhance uptake. Overall, they emphasized the need for consistent processes and procedures across institutional ICUs to support sustained implementation of the GTKMB. Participants identified patients who are unable to speak for themselves as those most likely to benefit from the GTKMB. They also emphasized the risk of the GTKMB becoming a checklist task, which could undermine one of its main values-supporting authentic human interactions. Physicians recommended integrating the GTKMB into rounds and role-modeling its use for trainees. Key contextual elements influencing GTKMB adoption included patient and family characteristics, the tool’s accessibility, and clarity regarding responsibility. Other strategies included completing the GTKMB in the pre-operative period for elective surgical procedures and admissions and making the board transferable across diverse levels of care during the hospital stay. These findings align with prior research emphasizing the importance of context and stakeholder engagement in implementing evidence-based practices (24).

Research by Hoad et al. (25) in the Footprints Project highlighted value of a tool like the GTKMB for patients and clinicians in a Canadian ICU yet also identified implementation barriers similar to the ones we identified. These barriers included limited visibility of the intervention, restricted availability, low staff motivation, and lack of accountability for completing of Footprints form and whiteboard (25). While the value of personhood in medical care has been established through tools such as Patient Dignity Questions or the *This Is Me* questionnaire, patients have suggested that such information be included in their medical charts (26–29). Similarly, in our study, integrating the GTKMB into the institutional EHR was considered an important facilitator.

The implications of implementing the GTKMB successfully and consistently could include improved consideration of patient values and goals, enhanced shared decision making, and efforts to provide goal-concordant care as key components of patient and family centered care (30–33). Participants in this study highlighted the value of understanding patient’s lives outside the hospital. This knowledge helped clarify patient’s goals and informed clinical decision-making. However, including goals in the GTKMB was seen as controversial, partly because there are other systems, in the EHR and in-room white boards, where goals can be documented and revised. The need to frequently update goals makes using the GTKMB in its current form challenging for tracking of goals.

Communication is one of the eight core principles established underlying humanized care in the ICU (1, 2, 34, 35). However, evaluating interventions designed to support communication is challenging due to the lack of clearly defined meaningful versus measurable outcomes (36–39). Within the hierarchy of communication, establishing trust and emotional connection precedes the cognitive aspects of information exchange (40). The impact of how the GTKMB is leveraged on both the hierarchy of communication in the ICU as well as on key stakeholder outcomes has yet to be studied. Applying quality improvement and evidence-based practice implementation science frameworks to define context specific process and outcome measures represents an important next step to build on the insights garnered from this study.

Limitations of this study include being conducted at a single center academic institution, which may limit the generalizability of our findings, as barriers, facilitators, and implementation strategies could differ in other settings. Additionally, participants who chose to take part may hold strong opinions about the GTKMB, which may not reflect the perspectives of non-participating clinicians. A specific implementation framework was not used to frame the study since the context was not in relation to a specific implementation initiative.

Strengths of our study include the recruitment of multidisciplinary ICU providers, allowing for triangulation of perspective, and the use of rigorous qualitative methods, with each transcript independently reviewed and coded by two researchers experienced in qualitative methods.

Although this study was not designed to evaluate implementation strategies, the results have led to institutional initiatives to promote use of the GTKMB. This work provided useful insights for conducting a quality improvement initiative in our medical ICU resulting in substantial increase in completion of the GTKMB in the ICU. This initiative has sparked institutional interest in exploring the integration of the GTKMB into the existing EHR platform. Furthermore we are currently conducting research to evaluate the accuracy and reliability of a multilingual, artificial intelligence- based tool to facilitate GTKMB completion, supporting broader implementation (41). Consistent adoption of the GTKMB within ICU workflows will be essential before larger studies can be conducted to evaluate meaningful patient outcomes.

## Conclusion

This study builds on prior work demonstrating the benefits of the GTKMB in promoting humanizing care. Here, we focused on factors that influence consistent use of the GTKMB. Although the GTKMB is recognized as an effective intervention for supporting person-centered care in the ICU, barriers to its consistent use remain. Strategies to facilitate adoption include role modeling, ensuring the board is accessible, fostering shared accountability for its completion, and emphasizing its use with the right patient at the right time.

## Data Availability

The original contributions presented in the study are included in the article/Supplementary material, further inquiries can be directed to the corresponding author.
