# Supplementary material for: Humanizing care in the ICU: implementation of the Get to Know Me Board in patient care
Source: Front Med (Lausanne). 2026 Mar 31;13:1796720. doi: 10.3389/fmed.2026.1796720 (PMC13076295; doi:10.3389/fmed.2026.1796720)
Supplement: Supplementary file 1 [file Supplementary_file_1.docx]

**Appendix 1**

**Focus group discussion guide:**

1. What does it mean to see the human side of a critically ill patient to you? (how does it translate to you clinical practice)
2. What aspects of the human side of the critically ill patient are important for you?
3. At current time, this is how the GTKM board looks (show the board). (Does this information help you interact with patient and his/ her family? Would there be features important to you to know, that you wished would be there? give examples)
4. So we have this tool, the GTKMB (showing the picture or poster of it) in the ICUs. Has this tool been helpful to you in your practice or not really? (How, examples?)
5. What are some of the barriers you see in incorporating the GTKM board in your clinical practice? (give examples).
6. Would there be times, you may hesitate or feel afraid of knowing the patient?
